# The preparation of oleylamine modified micro-size sphere silver particles and its application in crystalline silicon solar cells

**DOI:** 10.1039/c8ra02620c

**Published:** 2018-05-08

**Authors:** Feng Lan, Jintao Bai, Hui Wang

**Affiliations:** Key Laboratory of Synthetic and Natural Functional Molecule Chemistry (Ministry of Education), College of Chemistry & Materials Science, Northwest University Xi'an 710069 P. R. China huiwang@nwu.edu.cn +86 29 8830 3798 +86 29 8836 3115; National Key Laboratory of Photoelectric Technology and Functional Materials (Culture Base), National Photoelectric Technology and Functional Materials & Application of International Science and Technology Cooperation Base Institute of Photonics & Photon-Technology, Northwest University Xi'an 710069 P. R. China jintaobai@sina.cn baijt@nwu.edu.cn +86 29 8830 3798 +86 29 8830 3877

## Abstract

In this paper, micro-sized silver particles were prepared using a simple chemical approach without adjusting the pH of the solution, and oleylamine as a capping agent was described to promote the dispersion of the silver particles. The effects of temperature, feeding time and stirring speed on the morphology and size distribution of silver particles were discussed. The optimal reaction parameters (35 °C, 45 min, 300 rpm) for large scale industrial production were selected to obtained optimal silver particles for preparation of the silver paste for poly-crystalline silicon solar cells. The photoelectric conversion efficiency of the solar cells made with silver paste by using the silver particles can reach 18.623%, which is similar to the industrial production of solar cell made with commercial silver paste. This work contributes to the development of the synthesis of highly disperse silver particles used in solar cells in large scale industrial productions.

## Abstract 2

In this paper, micro-sized silver particles were prepared using a simple chemical approach without adjusting the pH of the solution, and oleylamine as a capping agent was described to promote the dispersion of the silver particles.

## Graphical abstract

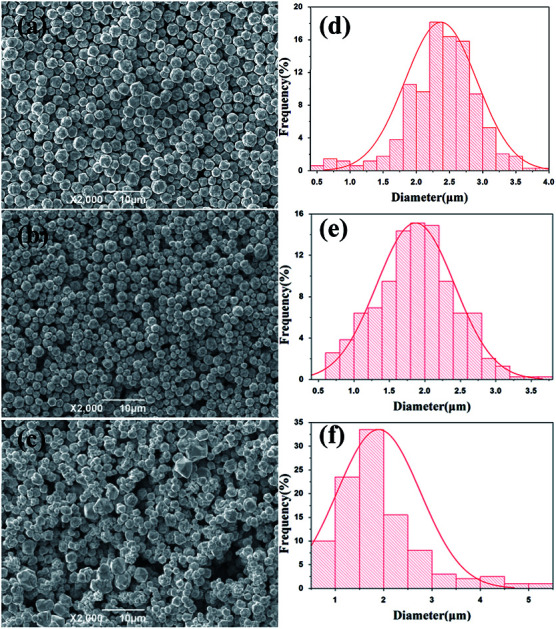

## Introduction

The development of solar cells provided the technology to use solar energy, converting solar energy directly into electric energy using the photovoltaic effect.^1^ Solar cells fall into several classes based on the materials they are made from: silicon solar cells, multi-compound film solar cells, organic solar cells, nano-crystalline solar cells and plastic solar cells.^2–9^ Of these, silicon solar cells have been used in industrial production due to the advanced technology and high conversion efficiency.^10^ To date, silver conductive thick films formed by screen printing are commonly used for the metallization contacts of crystalline silicon solar cells.^11–13^ The front electrode paste of silicon solar cells is composed of three main components, silver particles (conductive phase), glass powders (binder phase) and organic medium (disperse phase).^14–16^ The weight percent of silver particles in the electronic paste is greater than 80%. Therefore, the properties of silver particles are the critical factors in inducing conductivity and compactness of the front grid electrode.^17^ The optimal electronic paste requires the specific silver particles for crystalline silicon solar cells. In general, the shape of the silver particles for crystalline silicon solar cells is spherical. The silver paste used for crystalline silicon solar cells possess a lower porosity, higher open circuit voltage (*V*_OC_) and fill factor (FF) after the screen printing and sintering processes.^18^ The size distribution is also a critical factor in the efficiency of photoelectric conversion with the ideal size distribution being approximately 1–2 μm, the narrow size distribution can make silver paste uniform sintering and fewer pores in its fired film during high temperature firing, which will cause low contact resistance.^19^ The dispersion of silver particles is another important factor which effects the performance of the electronic paste, thus affecting the photoelectric conversion efficiency of solar cells.^20^ Therefore, the reaction conditions is important to guide the synthesized of silver particles. In the past few decades, focus on solar energy and electronic paste has been extensive, with many papers describing the synthesis of micro-sized silver particles being reported in the literature, and the effects of disperser agents, pH values, reducing agents, reaction temperature and feeding time discussed.^21–23^

There are numerous methods described to prepare micro-sized silver particles, such as water atomization,^24^ spray pyrolysis,^25^ electrolysis,^26^ chemical methods *etc.*^27^ Chemical methods are widely used in large scale industrial production due to the low cost, simple process and easy control.^28^ For large scale industrial production, ascorbic acid is used as the reducing agent because of its gentle reducibility and is environmentally friendly.^19^ In order to acquire the appropriate silver particles for crystalline silicon solar cells, we chose silver nitrate as the silver source, ascorbic acid as the reducing agent and poly(vinyl alcohol) as the dispersing agent in our study. In this study, oleylamine used as a capping agent was first introduced to promote the dispersion of micro-sized silver particles, and the interaction between oleylamine and silver particles was discussed. The effects of temperature, feeding time and mixing speed on the morphology and nucleation of silver particles was investigated. The particles morphology and structure were characterized using a Laser Particle Size Analyzer (LPSA), Fourier Transform Infrared Spectroscopy (FTIR), Thermal Gravimetric Analyzer (TGA), X-ray diffraction (XRD) and Scanning Electron Microscope (SEM). The particles were used for the preparation of silver paste used in poly-crystalline silicon solar cell to achieve superior aspect ratio and high conversion efficiency.

## Experimental

### Materials

Silver nitrate (AgNO_3_), ascorbic acid (C_6_H_8_O_6_), poly(vinyl alcohol) (PVAL), oleylamine (C_18_H_37_N) were all A.R. Grade and all of the reagents were purchased from Sinopharm Chemical Reagent Co. Ltd. Ultrapure water (*ρ* = 18.25 MΩ) was used in all experiments.

### Synthesis of micro-sized silver particles

In a typical synthesis process, two dispersing agents were used to synthesize the silver particles, and PVAL (the mass ratio of PVAL/AgNO_3_ = 0.8) and oleylamine (the mass ratio of oleylamine/AgNO_3_ = 0.05) were added to ascorbic acid solution (AA, 1.2 mol L^−1^). Firstly, the PVAL–AA solution was heated to specific temperatures (20 °C, 35 °C, and 50 °C) while being mechanically stirred at a specific speed (200 rpm, 300 rpm, and 400 rpm), then the preheated AgNO_3_ solution (1.55 mol L^−1^) was slowly injected into the PVAL–AA mixture solution using a peristaltic pump (leadfluid BT101L) at a specified feeding time (25 min, 45 min, 65 min) and continued to stirred for 1 h. After precipitate formed, the silver particles were washed using ultra-pure water five times, to remove the excess of PVAL, and then dried at 90 °C for 2 h.

### Measurements

Laser Particle Size Analyzer (BT-9300S, Dandong city Baxter instrument Co. Ltd.) was used to measure the size distribution of silver particles. X-ray diffraction (XRD) was used to determine the crystal structure of the silver particles, using a diffraction meter (D/Max-3C) and Cu-Kα radiation (*k* = 0.54184 nm) at a tube voltage of 45 kV and tube current of 200 mA. Fourier Transform Infrared Spectroscopy (FTIR) was carried out to determine the nature of the bonding with the surface of the silver particles using the KBr technique. Thermal Gravimetric Analyzer (STA-449C) at a heating rate of 10 K min^−1^. 3D microscopy (QUESTAR, RH-2000) was used to determine the morphology and aspect ratio of the front electrode grid line of poly-crystalline silicon solar cells. The photoelectric properties of polycrystalline silicon solar cells were measured using an EKO I-V Tracer under standard testing conditions (STC): solar radiation of 1000 W m^−2^ at 25 °C.

## Results and discussion

### Effects of temperature on the size distribution and morphology of silver particles

**Fig. 1:**
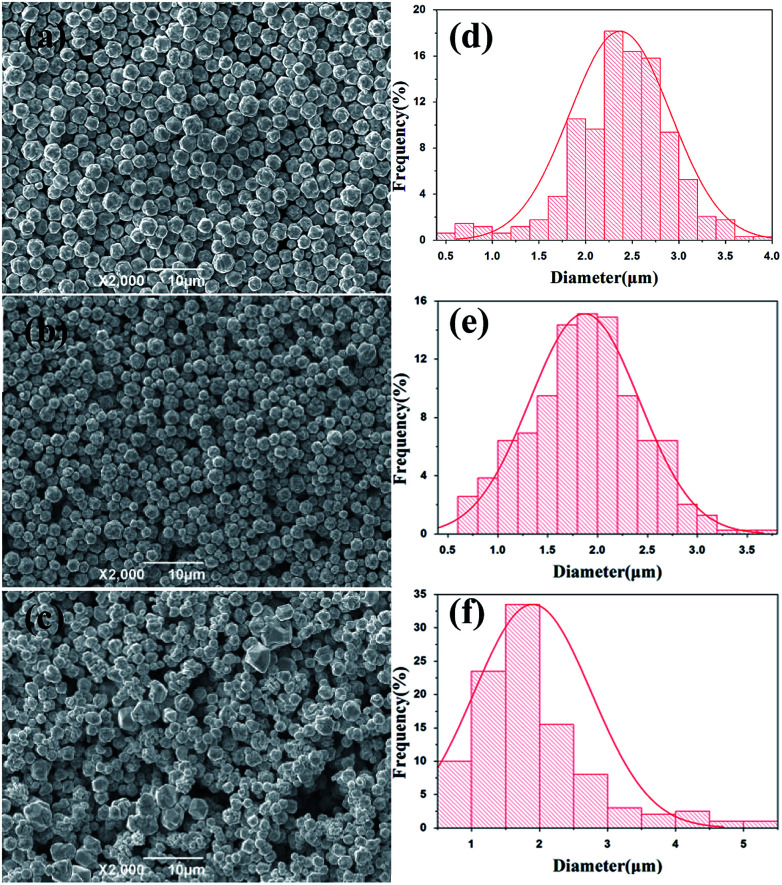
SEM images of silver spherical particles at different temperatures (a) 20 °C; (b) 35 °C, (c) 50 °C; size distribution of silver particles at different temperatures (d) 20 °C; (e) 35 °C, (f) 50 °C.

For large scale industrial production in aqueous solution, the temperature is a vital factor in determining the size and shape of the silver particles. The reaction temperature should be easy to control. The effects of reaction temperature on morphology and size distribution of silver particles was studied, and the feeding time (45 min) and stirring speed (300 rpm) were selected for experiments. Fig. 1(a–f) shows representative SEM images and the size distribution of spherical silver particles prepared at various temperatures (20 °C, 35 °C, and 50 °C). It can be observed that with the increase of temperature from 20 °C to 35 °C, the percentage of small silver particles increases and large silver particles decreases. The size distribution of silver particles is seen in Fig. 1(d and e), which is consistent with Fig. 1(a and b). When the temperature was increased from 35 °C to 50 °C, the degree of crystallization of the spherical silver particles became greater, and which was demonstrated in the XRD data. The size of the silver particles size showed a increasing trend as temperature increased, some large and irregular particles were found at higher temperatures and the size distribution of silver particles was wide (Fig. 1c and f). This could be due to Brownian motion, collision of small particles and agglutination between particles as the temperature increased.^29^ The results indicated that temperature has a significant impact on the morphology of the silver particles, as the temperature increased, the diameter of the silver particles decreased wholly, but at higher temperature, some large particles appeared due to the faster reaction rate. Compared to the temperatures (20 °C), the silver particles synthetized were smaller and uniformly at the higher temperature (35 °C), and it was suitable for industrial production.

### Effects of feeding time on the size distribution and morphology of silver particles

**Fig. 2:**
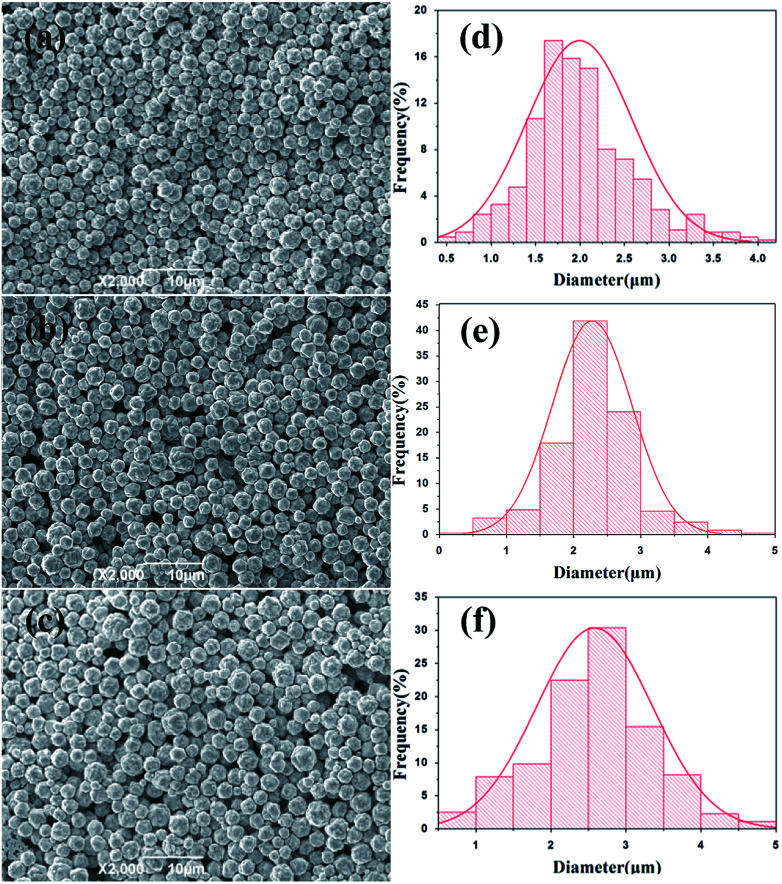
SEM images of silver spherical particles at different feeding time (a) 25 min; (b) 45 min, (c) 65 min; size distribution of silver particles at different feeding time (d) 25 min; (e) 45 min, (f) 65 min.

To determine the effects of feeding time on morphology and size distribution of the silver particles, different feeding times (25 min, 45 min, 65 min) were examined, while the reaction temperature (20 °C) and stirring speed (300 rpm) remained constant. The SEM images of silver particles are seen in Fig. 2(a–c), the size distribution of the silver particles are present in Fig. 2(d–f). When time is increased from 25 min to 45 min, the diameter of silver particles increased and the size distribution became narrower. When the feeding time was increased from 45 min to 65 min, the silver particle size continued to increase and the size distribution became wider. Small particles were observed under different reaction conditions. Kwon and Hyeon^30^ described the influence of the degree of super-saturation on the nucleation rate, and demonstrated that the nucleation rate increased with the increase of degree of super-saturation. When feeding time was extended, the super-saturation of bulk solution was reduced in unit time, the nucleation rate went down and the diameter of the silver particles increased. What's more, Ostwald ripening also caused the diameter of silver particles increased.

### Effects of stirring speed on the size distribution and morphology of silver particles

**Fig. 3:**
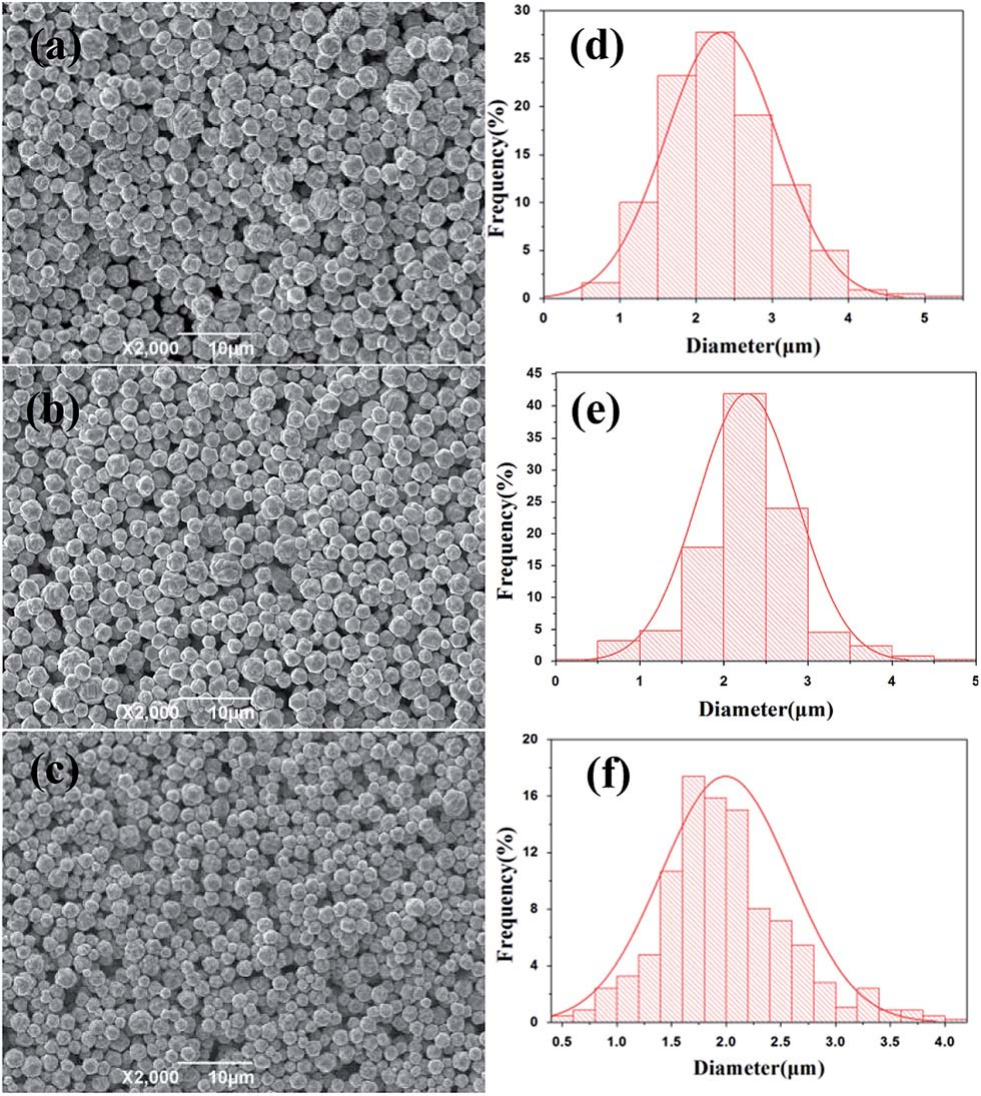
SEM images of silver spherical particles at various stirring speed (a) 200 rpm; (b) 300 rpm, (c) 400 rpm; size distribution of silver spherical particles at various stirring speed (d) 200 rpm; (e) 300 rpm, (f) 400 rpm.

To examine the effects of stirring speed on the morphology and size distribution of silver particles, a reaction temperature (20 °C) and time (45 min) were selected for all experiments, and a set of experiments were carried out to described the relationship between the size distribution and the stirring speed. Fig. 3(a–c) show representative SEM images of the spherical silver particles prepared at various stirring speeds. Fig. 3(d–f) shows the size distribution of the spherical silver particles prepared at various stirring speeds. As shown in Fig. 3a, when the stirring speed was 200 rpm, size distribution of silver particles was wide, and with significant numbers of large and small particles. This could be due to the coagulation growth of small particles. Furthermore, when the stirring speed was increased, the number of larger and small particles gradually disappeared, and the size distribution of silver particles became uniform (Fig. 3(b, c, e and f)). The results indicate that stirring speed has a significant impact on the size distribution of the silver particles, and the size distribution of the silver particles synthesized with the stirring speed (400 rpm) was more uniform compared to the stirring speed (300 rpm). The stirring speed should be considered carefully, so as to not lose solution during stirring, at higher stirring speeds. Therefore, a stirring speed of 300 rpm was chosen for large scale industrial production.

### Growth process and analyses of micro silver particles

**Fig. 4:**
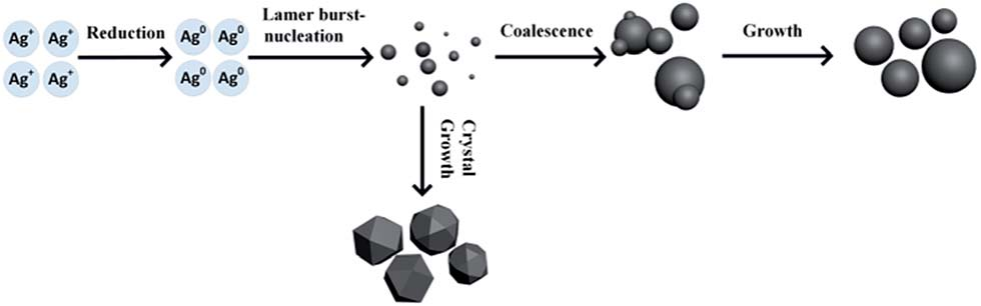
Schematic illustration of the growth process of micro-sized silver particle.

As described above, the effects of diverse reaction conditions on the size distribution and morphology of silver particles were studied. The results indicated that the reaction temperature has a significant influence on the morphology and degree of crystallization of silver particles, and increased feeding times and selected stirring speeds contribute to producing uniform sized silver particles. In order to develop optimal silver particles for crystalline silicon solar cells, the reaction parameters 35 °C, 45 min, 300 rpm were selected. The different time size distributions and morphology of the silver particles were monitored during the preparation of silver particles using laser particle size analyzer and SEM, respectively. The synthesis of ultra-micro particles in the solution requires two steps: nucleation and growth of particles. According to the classical nucleation theory, stable particles within a solution can be obtained when free energy reaches the critical value, *i.e.* the super-saturation of the solution is sufficient to overcome the nucleation potential which a particle can survive in solution without being redissolve.^31^ In general, crystalline growth process of particles consists of crystal growth and coagulation growth.^32^ However, for the synthesis of ultra-micro particles in the solution, coalescence and aggregation is the basic principles of particle growth, which is due to the monomer-supplying chemical reaction is faster than the actual particle formation.^33^ A schematic illustration of the growth process of micro-sized silver particles is seen in Fig. 4. Initially, the silver nitrate solution was injected into the reduction solution, and free monomers increased rapidly in solution. The monomers then undergo burst-nucleation while the supersaturation of the solution reaches critical level. Finally the crystal nuclei undergo coalescence and aggregation, and form the final particles.

**Fig. 5:**
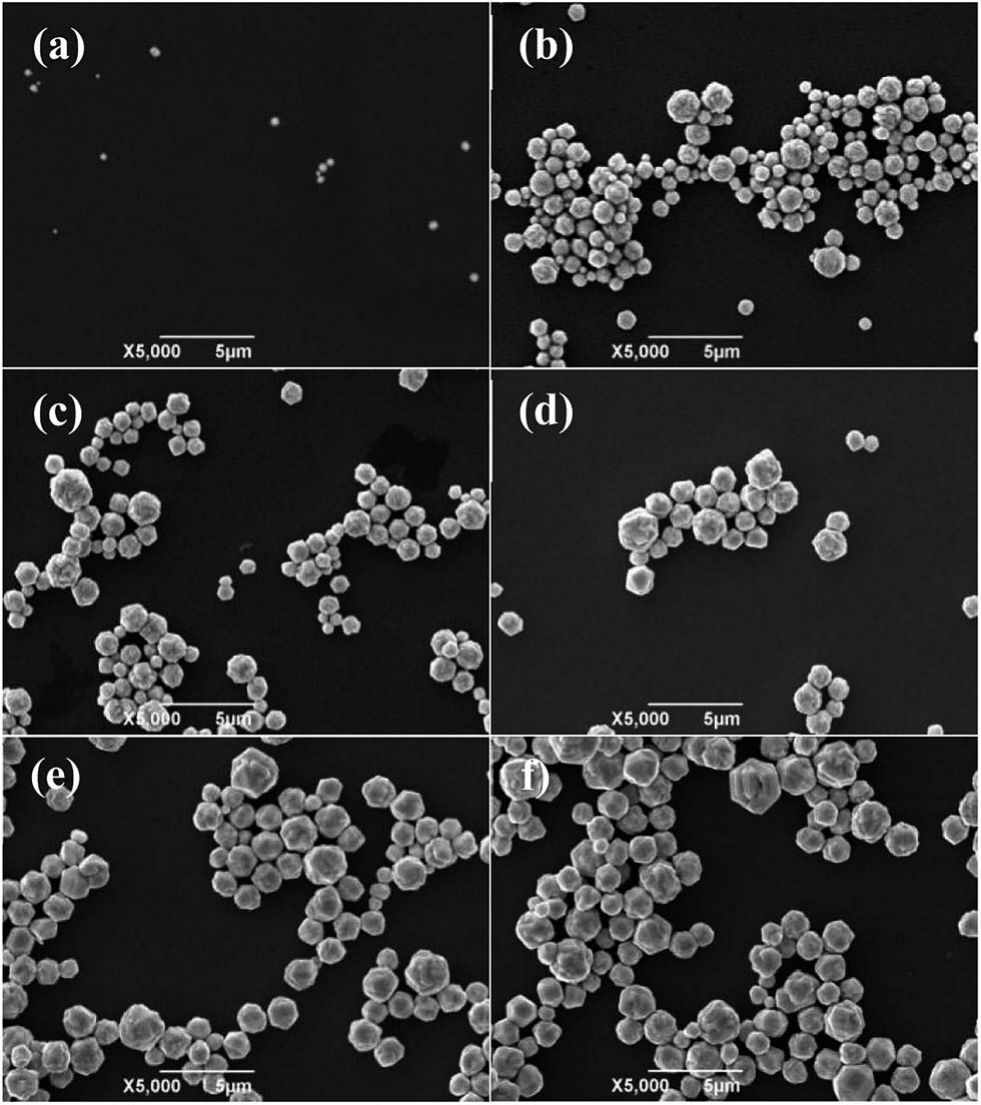
SEM images of silver particles at the various reaction times during the reaction process: (a) 1 min; (b) 10 min; (c) 20 min; (d) 30 min; (e) 45 min; (f) 55 min.

**Fig. 6:**
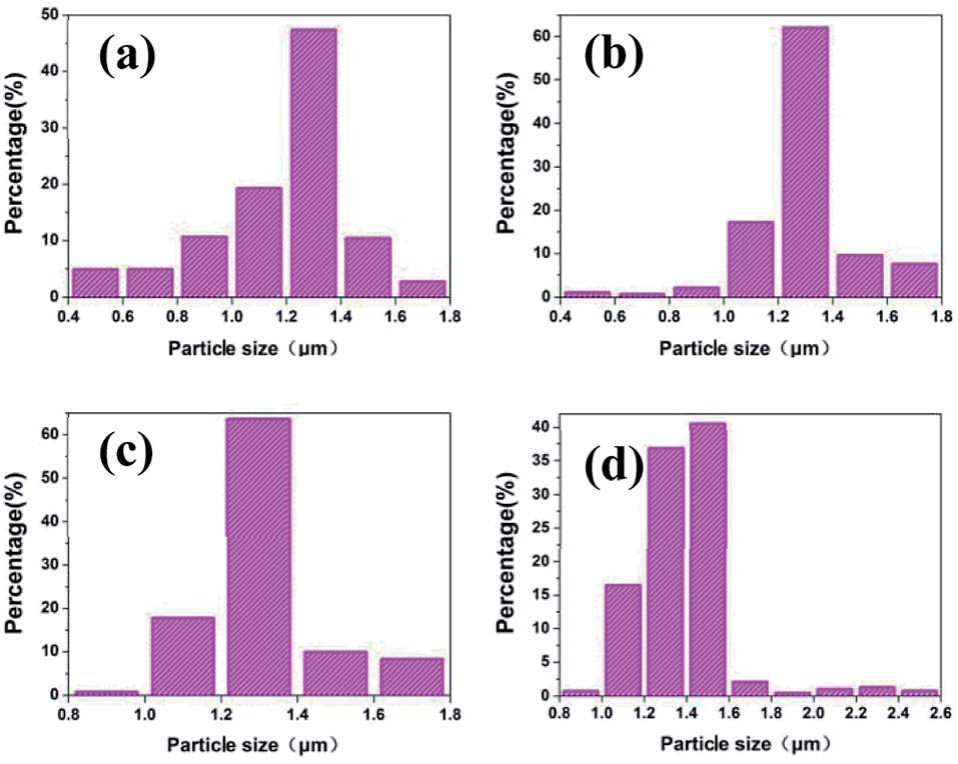
Size distribution of silver particles synthesized at the various feeding times during the reaction process: (a) 10 min; (b) 20 min; (c) 30 min; (d) 45 min.

Fig. 5(a–f) shows the variation in SEM images and size distribution of silver particles as determined using a laser particle size analyzer from the reaction mixture at the different reaction times. It can be observed that the D50 of the silver particles was 1.3 μm at the reaction times 10 min, 20 min and 30 min measured by the laser particle size analyzer. Moreover, when the reaction is increased from 10 min to 30 min, the number of small particles was reduced and the number of larger particles was increased (Fig. 5(b–d)). With further reaction, the silver particles D50 reached 1.5 μm, and the size of the largest particles reached 2.5 μm, also presented in the SEM Fig. 5e. When the reaction ended, the solution continued to be stirred, and the size distribution of the silver particles remained similar, with the silver particles D50 1.5 μm (Fig. 5f and 6).

### XRD patterns

**Fig. 7:**
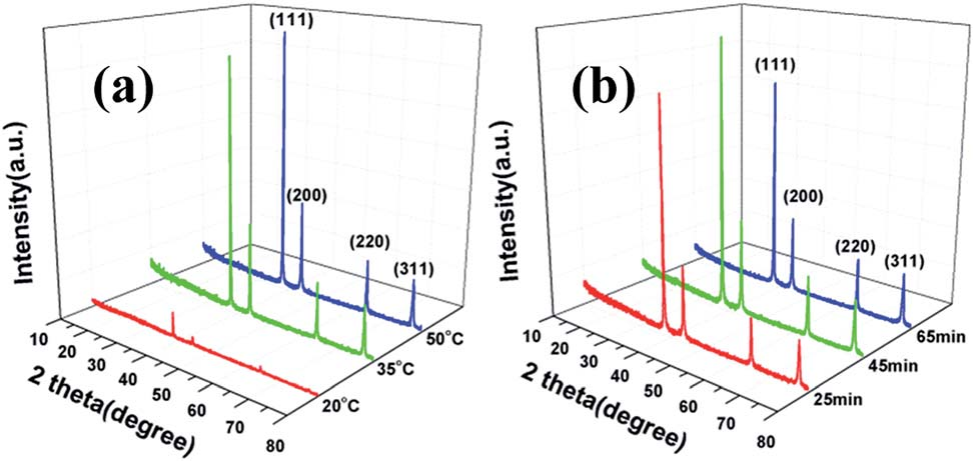
XRD patterns of silver particles (a) different temperatures; (b) various feeding times.

The effects of reaction temperature and time on morphology and size distribution of silver particles was studied, with X-ray diffraction (XRD) used to measure the crystal structure of the silver particles. As shown in Fig. 7, all the peaks in the XRD patterns are in accordance with standard crystalline silver cards (JCPDS card no. 04-0783) and it can be seen that the reaction temperature had a significant influence on the crystallinity of the silver particles, *i.e.* the higher the temperature, the greater the degree of crystallization. The feeding time had little impact on the crystallinity of the silver particles, as observed Fig. 7b.

### TGA

**Fig. 8:**
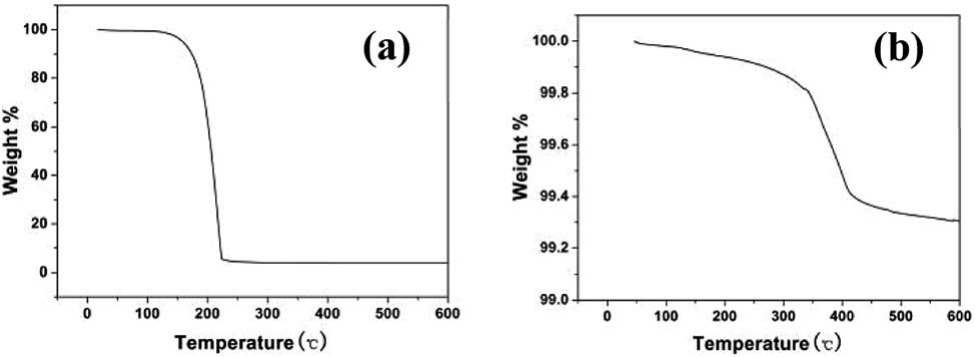
TGA curves of (a) free oleylamine; (b) oleylamine modified silver particles.

To determine the interaction between oleylamine and silver particles, TGA was carried out under nitrogen gas. Fig. 8 shows the TGA curves of free oleylamine (a) and oleylamine modified silver particles (b). A significant loss of free oleylamine at about 180 °C was observed in Fig. 8a, while the TGA curves of oleylamine modified silver particles (Fig. 8b) indicated a decreasing trend. A broad weight lose curve in Fig. 8b were seen at approximately 340 °C, and the temperature of weight loss is greater than that of free oleylamine. It may be that some chemical bonds formed between the dispersant (oleylamine) and the surface of the particle. This agrees with that reported in the literature, which describes the weight loss at higher temperature is due to chemically bound surfactant on the surface of the particle.^34^

### FTIR spectra

**Fig. 9:**
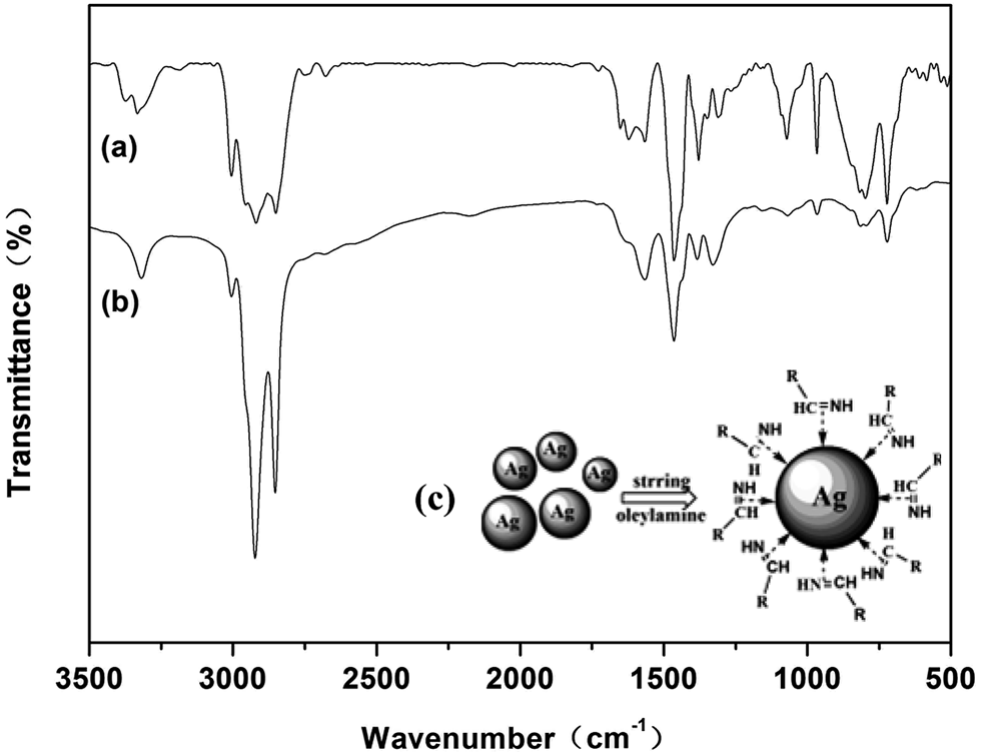
FTIR spectra of (a) free oleylamine; (b) oleylamine modified silver particles; (c) schematic illustration of the oleylamine modified silver particles.

FTIR spectra was carried out further to understand the nature of the bonding of oleylamine with the surface of the silver particles. Fig. 9 shows the spectra of free oleylamine and oleylamine modified silver particles. As can be observed in the FTIR spectra of free oleylamine (similar to that previously reported^35,36^), the bands at 3373 and 3333 cm^−1^ are due to *ν*_as_(NH_2_) and *ν*_s_(NH_2_) vibration modes, the bands at 2850 and 2920 cm^−1^ are due to the *ν*_as_(CH_3_) and *ν*_as_(CH_2_) vibration modes, the band at 3004 cm^−1^ is attributed to =C–H stretching vibration, the bands at 1566 and 794 cm^−1^ are attributed to NH_2_ bending vibrations, and the absorption bands at 1651, 1464, 1072 and 722 cm^−1^ correspond to –C=C bending vibrations, CH_3_ bending vibrations, C–N bending vibrations and C–C bending vibrations, respectively.

It is seen that the positions of the peak frequencies of oleylamine modified silver particles are similar to that of the free oleylamine. Compared with the spectra of free oleylamine, the absorption bands of *ν*_as_(CH_3_) and *ν*_as_(CH_2_) of oleylamine modified silver particles are consistently red-shifted by 2 cm^−1^: *ν*_as_(CH_3_) = 2922 cm^−1^, *ν*_as_(CH_2_) = 2852 cm^−1^. *δ*(NH_2_) was shifted to 1565 and 796 cm^−1^. Furthermore, *δ*(=C–H), *δ*(CH_3_), *δ*(C–N) and *δ*(C–C) were shifted to 3318 cm^−1^, 1463 cm^−1^, 1072 cm^−1^ and 721 cm^−1^, respectively. These slight shifts have been reported by others in the literature, and it could be caused by the formation of a layer with the oleylamine which caps the silver particles.^37^ The largest variation between the free oleylamine and the oleylamine modified silver particles in the FTIR spectra was the double peak of the *ν*_as_(NH_2_) and *ν*_s_(NH_2_) vibration modes transformed into the single peak at 3320 cm^−1^, which may be caused by the stretching vibration of the imines. The bands at 2177 cm^−1^ also suggest the presence of a imines.^38^ A schematic of the oleylamine modified silver particles in the presence of C=N is presented in Fig. 9c. The results of the TGA and FTIR spectra indicated that the imines formed after the silver particles formed. The lone pair electrons on the π-system of the double bond coordinated with the surface of silver particles, which can passivate the surface of silver particles, thus, preventing the agglomeration of the silver particles and promoting the dispersion of silver particles.

### Application

The silver paste (SP1) was prepared by mixing silver particles and organic medium^39^ (prepared by our experiment) and glass powders^40^ (prepared by our experiment) at a ratio of 85/5/10 (wt%) using a roller machine (Puhler, PTR65C). Poly-crystalline silicon solar cells were produced by screen printing the silver paste (SP1) and commercial silver paste (SP2) on the front side of p-type poly-crystalline silicon wafers (156 × 156 cm) with an emitter approximately 60 Ω sq^−1^, which were dried in an air oven for 1 h at 50 °C.

**Fig. 10:**
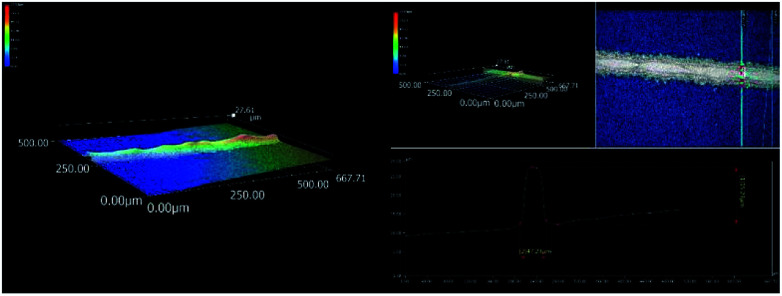
3D microscopy of the morphology of the front electrode grid line of poly-crystalline silicon solar cell.

The poly-crystalline silicon solar cells made from SP1 were denoted as SC1, SC2 and SC3, and those made from SP2 were denoted as SC4 and SC5. Finally, the electrical performance parameters were measured using an in-line furnace (Table 1). The photoelectric conversion efficiency of the poly-crystalline solar cell fabricated with the synthesized silver particles could reach 18.636%, which is similar to commercial products. The morphology of the front electrode grid line were determined using a 3D microscope, Fig. 10, the front electrode grid line of poly-crystalline silicon solar cells had a good aspect ratio.

**Table 1:** Electrical performance parameters of solar cells SC1, SC2, SC3, SC4 and SC5

Paste	Cell	*V*_OC_ (V)	*I*_SC_ (A)	*V*_mp_ (V)	*I*_mp_ (A)	*P*_mp_ (W)	*R*_S_ (Ω)	*R*_Sh_ (Ω)	FF (%)	Eff (%)
SP1	SC1	0.637	9.014	0.537	8.519	4.579	0.0018	63.660	79.798	18.636
SP1	SC2	0.638	8.977	0.540	8.469	4.576	0.0015	118.899	79.887	18.623
SP1	SC3	0.638	9.007	0.539	8.495	4.575	0.0020	280.584	79.633	18.621
SP2	SC4	0.636	8.980	0.540	8.485	4.580	0.0014	236.837	80.239	18.639
SP2	SC5	0.637	8.976	0.540	8.481	4.578	0.0015	204.481	80.052	18.632

## Conclusions

In this study a simple chemical approach was demonstrated to synthesize highly disperse micro-size silver particles without adjusting the pH of the solution, and oleylamine was first introduced to promote the dispersion of micro-sized silver particles. The effects of temperature, feeding time and stirring speed on the morphology and size distribution of silver particles were discussed. The results indicate that the reaction temperature is a significant factor in controlling the morphology and crystallinity of silver particles, which was confirmed by SEM images and XRD patterns. We also suggest that uniform silver particles can be obtained by prolonging the feeding time and selecting appropriate stirring speeds. Optimal reaction parameters of 35 °C, 45 min and 300 rpm for large scale industrial production were selected to produce suitable silver particles for the preparation of the silver paste for the use in poly-crystalline silicon solar cells. The solar cell made with silver particles synthesized under optimal reaction parameters show superior aspect ratio and high photoelectric conversion efficiency (18.636%), which is similar to solar cells (18.639%) made with commercial silver pastes. This work contributes to the development of the synthesis of highly disperse silver particles used in solar cells in large scale industrial productions. We believe that the improvement of the technology and performance of the glass powders and organic medium will further promote the photoelectric conversion efficiency.

## Conflicts of interest

There are no conflicts to declare.

## Supplementary Material
